# Decline in US Drug Overdose Deaths by Region, Substance, and Demographics

**DOI:** 10.1001/jamanetworkopen.2025.14997

**Published:** 2025-06-12

**Authors:** Lori Ann Post, Daniel Ciccarone, George Jay Unick, Gail D’Onofrio, Soyang Kwon, Alexander L. Lundberg, Shivangi Sharma, Maryann Mason

**Affiliations:** 1 Department of Emergency Medicine, Northwestern Feinberg School of Medicine, Chicago, Illinois; 2 Buehler Center for Health Policy and Economics, Northwestern Feinberg School of Medicine, Chicago, Illinois; 3 Family and Community Medicine, University of California, San Francisco; 4 School of Social Work, University of Maryland, Baltimore; 5 Department of Emergency Medicine, Yale School of Medicine, New Haven, Connecticut

## Abstract

**Importance:**

Drug overdose deaths (DODs) surged with the advent of fentanyl. Recent US reports indicated a decline, but standard surveillance systems do not account for monthly variability or seasonality and require monthly population data to calculate DOD rates.

**Objective:**

To identify when US DOD rates began to decelerate and to examine patterns by census region, drug type, and demographics.

**Design, Setting, and Participants:**

This repeated cross-sectional study of DOD rates was conducted from January 2015 to October 2024, using data from the National Center for Health Statistics and US Census Bureau. Decedents included those whose drug poisoning death was classified as unintentional, intentional (suicide or homicide), or undetermined intent, identified by *International Statistical Classification of Diseases and Related Health Problems, 10th Revision* codes for external overdose causes and T codes for opioids, cocaine, and psychostimulants (eg, methamphetamine).

**Main Outcomes and Measures:**

The main outcome was change in monthly DOD rates nationally and by drug type (opioids, cocaine, or methamphetamine), census region, and demographics. Joinpoint regression evaluated significant shifts in DOD rates applying the weighted bayesian information criterion and 2-sided *z* tests (α = .05).

**Results:**

A total of 800 645 US residents (68.3% male; median age, 42 years [IQR, 33-54 years]) died of drug overdose between January 2015 and October 2024. The national DOD rate increased from 14.54 (95% CI, 14.52-14.55) per 100 000 population in January 2015 to 33.24 (95% CI, 33.15 to 33.33) per 100 000 population in August 2023. From August 2023 to February 2024, the monthly DOD rate declined by −0.36 (95% CI, −0.46 to −0.27) per 100 000 population, accelerating to −0.84 (95% CI, −0.77 to −0.92) per 100 000 population through October 2024 and reaching 24.29 (95% CI, 24.21-24.37) per 100 000 population. Opioid-related DOD rates declined faster than stimulant-related DOD rates (−0.80 [95% CI, −0.74 to −0.87] vs −0.25 [95% CI, −0.23 to −0.27] per 100 000 population). While the national DOD rate peaked in August 2023, rates peaked in the Northeast, Midwest, and South census regions in October 2022 and the West peaked a year later. By late 2023, death rates continued to accelerate among adults aged 55 years or older (0.07 per 100 000 population) and American Indian or Alaska Native (0.02 per 100 000 population), Black or African American (1.70 per 100 000 population), Hispanic or Latino (0.20 per 100 000 population), and multiracial (0.28 per 100 000 population) populations, though the pace of increase was slowing, suggesting a potential inflection point.

**Conclusions and Relevance:**

In this cross-sectional study, US DOD rates entered a new wave of sustained deceleration in 2023 after 2 decades of increase. This shift may reflect changes in drug markets, treatment access, harm reduction efforts, and population-level risk. Although the decline is encouraging, persistent disparities highlight the need for targeted interventions and improved understanding of the underlying drivers.

## Key Points

**Question:**

When did US drug overdose deaths (DODs) begin to decelerate, and was that decline uniform across substances and demographics?

**Findings:**

In this cross-sectional study of 800 645 DODs, beginning August 2023, DODs declined for 15 consecutive months, with the largest decreases in the final 9 months of decline, although adults aged 55 years or older and American Indian or Alaska Native, Black or African American, Hispanic or Latino, and multiracial individuals had year-over-year increases. Opioid-related deaths decelerated faster than stimulant-related deaths.

**Meaning:**

While overdose deaths rapidly decelerated, the trend was neither uniform nor consistent across demographics or drug types.

## Teaser

This cross-sectional study investigates when the recent declines in US drug overdose death rates began and whether rate changes differed by census region, drug type, and decedent demographics.

## Introduction

The US annual drug overdose death (DOD) rate doubled between 2015 (16.25 per 100 000 population) and 2023 (32.76 per 100 000 population), contributing to a decline in life expectancy.^1,2^ To fully understand the drug overdose crisis, we must consider not only the number of deaths but also who is affected and where they live.^3,4^ Equally important is examining the central role that opioids have played as the primary driver of DODs.^5^ In 2022, opioids were involved in 76% of DODs; that share declined slightly to 75% in 2023 and further to 69% in 2024,^1^ a period when some state and federal agencies reported declines in overall DODs.^4,6,7,8^ In today’s drug overdose crisis, DOD rates have been shaped by increasingly toxic shifts in drug supply beginning with overprescribing opioid pain medications followed by overlapping waves of heroin, fentanyl, and polysubstance use.^9,10,11,12,13,14^ Despite the reports that DODs have declined recently, major questions remain. When exactly did the national decline begin? Was it consistent across US regions and demographic groups? Have there been meaningful changes in the drugs involved?

To detect emergent drug trends, monthly death estimates are more informative than annual data.^14^ While calendar year 2023 reflected the opioid crisis 1.5 years ago, monthly data offer a more timely signal. However, despite avoiding the long lags of annual reporting, monthly figures are inherently noisy. Annual totals, in contrast, can obscure seasonal fluctuations.^14,15^ Between January 1999 and October 2023, the monthly DOD rate ranged from 0.44 per 100 000 population (October 2000) to 2.8 per 100 000 population (July 2022).^1^ Over this 300-month span, the DOD rate declined 143 times, including 7 sustained decelerations lasting at least 3 consecutive months.^1^ The longest was a 5-month span from June to October 2000.^1^ What does it mean to say that there is now a decline in DODs when similar previous declines were noted 143 times? These patterns underscore the need for caution when interpreting short-term declines, which may reflect noise or seasonality rather than lasting change. It remains unclear how much of the reported 2024 decline represents true progress, statistical volatility, or a return to prepandemic trends.^16^ To that end, this study aimed to pinpoint when drug overdose trends entered sustained decline by addressing 3 evidence gaps: the absence of monthly population denominators to reduce death count–based noise, the need for annualized monthly estimates to adjust for seasonality, and the ability to differentiate plateaus and gradual trends from abrupt changes.

## Methods

In this cross-sectional study of DOD rates from January 2015 to October 2024, we standardized DODs by calculating death rates. We conducted joinpoint trend analyses to identify significant shifts in death rates over time for all drug, opioid, cocaine, and methamphetamine overdose deaths. We analyzed death rates across 4 US census geographic regions (Midwest, Northeast, South, and West) and by demographic characteristics. DODs were included if manner of death (underlying cause of death) was categorized according to the *International Statistical Classification of Diseases and Related Health Problems, 10th Revision (ICD-10)* codes X40 to X44 (unintentional [accidental] poisoning by drugs), X60 to X64 (intentional self-harm [suicide] by drug poisoning), X85 (intentional homicidal poisoning by drugs), and Y10 to Y14 (undetermined intent poisoning by drugs). *ICD-10* codes for external causes of death were paired with T codes for specific drugs, including opioids (T40.0-T40.4, T40.6), cocaine (T40.5), and psychostimulants, such as methamphetamine (T43.6). The study used publicly available, deidentified data and was determined exempt from approval and informed consent by the Northwestern University institutional review board. This study followed the Strengthening the Reporting of Observational Studies in Epidemiology (STROBE) reporting guideline.

### Data Sources

This study sourced data from the National Center for Health Statistics DOD tracking systems, Centers for Disease Control and Prevention WONDER databases, and National Vital Statistics System (NVSS)^1,17,18^ and US census monthly intercensal estimates and projections^19,20^ to calculate 12-month moving and calendar year death rates per 100 000 population to control for population growth, allowing comparison across time between demographic groups and geographic regions.^14,15^ Decedent demographics were derived from death certificates completed by medical examiners or coroners based on medical records, law enforcement reports, or direct observation. Racial groups included American Indian or Alaska Native, Asian, Black or African American, Native Hawaiian or Other Pacific Islander, White, and multiracial. Ethnic groups were Hispanic or Latino and not Hispanic or Latino.

### Statistical Analysis

#### Joinpoint Trend Analysis

Joinpoint trend analysis is a regression method used to identify inflection points (joinpoints) where trends shifted using permutation tests.^21^ In this study, analysis identified months with changes in DOD rate slope (independent variable) from January 2015 to October 2024. The best-fitting model was identified using the weighted bayesian information criterion, and slope significance was tested using 2-sided *z* tests (α = .05) on 118 months of data points. Analyses were repeated for DOD rates involving opioids, cocaine, and methamphetamine. Joinpoint analysis distinguishes between plateaus, gradual trends (inclines or declines), and abrupt changes (spikes or plummets) based on significant changes in slope. The joinpoint analysis was performed using Joinpoint Trend Analysis Software, version 5.3.0 (National Cancer Institute).

#### Epidemiologic Rate Trend Analysis

Descriptive rate trend analysis was conducted to quantify absolute and relative changes in DOD rates across US census regions and demographic subgroups. This method relied on repeated cross-sectional calculations of crude DOD rates per 100 000 population using 2 distinct temporal intervals, including nine 12-month annual sums ending on October 31 of each year from October 2015 (ie, including deaths occurring from November 1, 2014, through October 31, 2015) to October 31, 2024, and 9 calendar year aggregates from 2015 through 2023. Regional and demographic comparisons were used to examine the direction, magnitude, and pace of change over time, focusing on periods of acceleration or deceleration. This descriptive approach complemented the joinpoint analysis by offering a population-level perspective that contextualized statistically detected inflection points. Rate comparisons were tested for statistical significance using SPSS, version 29.0.0 (IBM Corp), and visualizations were created using R, version 4.2.2 (R Project for Statistical Computing).

## Results

Between January 2015 and October 2024, 800 645 US residents died of a drug overdose; 31.7% were female, and 68.3% were male, with median age of 42 years (IQR, 33-54 years).^1^ A total of 11.2% were Hispanic or Latino ethnicity, and 88.8% were not Hispanic or Latino. From 2018 through 2024, 1.4% of DOD decedents were American Indian or Alaska Native, 1.1% were Asian, 18.3% were Black or African American, 0.1% were Native Hawaiian or Other Pacific Islander, 77.6% were White, and 1.5% were multiracial. Breakdowns of data by racial group from 2015 to 2018 were excluded due to changes in racial classification by the US Census Bureau and the National Center for Health Statistics, rendering pre-2018 data incomparable.

**Figure 1.**
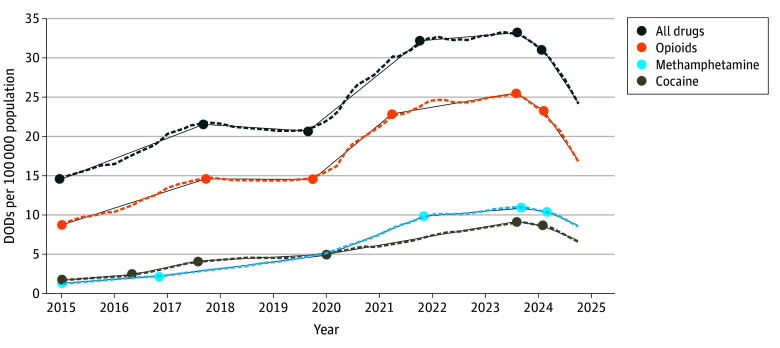
Joinpoint Analysis of Drug Overdose Death (DOD) Rates Based on data from the National Vital Statistics System and US Census Bureau intercensal population estimates.

The 12-month moving sum of DOD rates ranged from a low of 14.54 (95% CI, 14.52-14.55) per 100 000 population in January 2015 to a peak of 33.24 (95% CI, 33.15-33.33) per 100 000 population in August 2023. As shown in Figure 1, joinpoint regression analysis identified 5 significant inflection points in the trend. Between January 2015 and September 2017, monthly DOD rates increased steadily by 0.22 (95% CI, 0.20-0.24) per 100 000 population, reaching an inflection point at 21.57 (95% CI, 21.55-21.59) per 100 000 population in September 2017. From September 2017 to September 2019, rates plateaued, with a slight decrease (–0.04 [95% CI, −0.05 to −0.02] per 100 000 population), followed by a significant surge (0.46 [95% CI, 0.44-0.48] per 100 000 population) through October 2021, when the DOD rate reached 32.15 (95% CI, 32.13-32.17) per 100 000 population. A second rate plateau of 0.05 (95% CI, 0.03-0.07) per 100 000 population followed until August 2023, when the DOD rate reached 33.24 (95% CI, 33.15-33.33) per 100 000 population, representing a slight increase of 0.05 (95% CI, 0.03-0.07) per 100 000 population. From August 2023 to February 2024, DOD rates began to decline at a rate of –0.36 (95% CI, −0.46 to −0.27) per 100 000 population. In February 2024, a statistically significant shift occurred: the rate of decline more than doubled, accelerating to –0.84 (95% CI, −0.77 to −0.92) per 100 000 population, reaching 24.29 (95% CI, 24.21-24.37) per 100 000 population. This steeper decline continued through the most recent data point in October 2024. Notably, DOD rates decreased for 15 consecutive months, and the post–February 2024 deceleration was nearly twice the rate of the previous surge between 2019 and 2021 (0.46 [95% CI, 0.44-0.48] per 100 000 population).

Separate joinpoint models were constructed for each major drug type, producing 5 joinpoints. From 2015 to 2024, opioid-involved deaths were primarily driven by fentanyl and its analogues^22^ and closely mirrored the overall DOD rate trend, with similar shift points but differing magnitudes. The monthly opioid DOD rate began to decline in August 2023 (−0.36 [95% CI, −0.28 to −0.44] per 100 000 population), and the decline steepened in February 2024 (−0.80 [95% CI, −0.74 to −0.87] per 100 000 population). The monthly rate of cocaine-related deaths followed a similar pattern, declining from August 2023 (−0.07 [95% CI, −0.04 to −0.09] per 100 000 population) and decreasing further by February 2024 (−0.25 [95% CI, −0.23 to −0.28] per 100 000 population). Monthly methamphetamine-involved death rates began declining 1 month later, in September 2023 (−0.10 [95% CI, −0.07 to −0.12] per 100 000 population) followed by a steeper decline (−0.25 [95% CI, −0.23 to −0.27] per 100 000 population) in February 2024.

Methamphetamine displaced cocaine as the leading cause of stimulant-related overdose deaths in September 2019. While opioids accounted for a higher overall death rate than stimulants, their rate of DOD decline after August 2023 (−0.36 [95% CI, −0.46 to −0.27] per 100 000 population) was significantly faster than the rate for methamphetamine (−0.10 [95% CI, −0.12 to −0.07] per 100 000 population) after September 2023 and for cocaine (−0.07 95% CI [−0.09 to −0.04] per 100 000 population) after August 2023 (*P* < .001).

**Figure 2.**
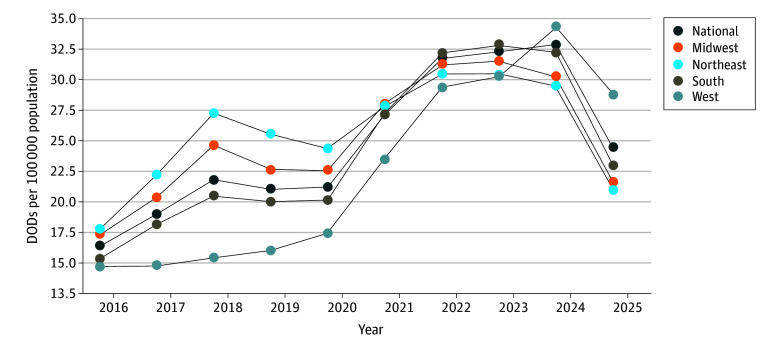
Annual Drug Overdose Death (DOD) Rates by US Census Region From October 2015 to October 2024 Based on data from the National Vital Statistics System.

Figure 2 illustrates differences in annual census DOD rates across US census regions ending on October 31 of each year since 2015. The West had the lowest DOD rate at 14.64 per 100 000 population in October 2015, while the Northeast had the highest at 17.27 per 100 000 population. Peaks in the Northeast (30.42 per 100 000 population), Midwest (31.40 per 100 000 population), and South (32.66 per 100 000 population) occurred earlier, in October 2022, compared with the national DOD rate peak (32.76 per 100 000 population) in October 2023. The West diverged from other regions, accelerating rapidly to 34.21 per 100 000 population by October 2023. Rates in the West subsequently declined, but it maintained the highest annual DOD rate of any region at 28.72 per 100 000 population in October 2024. By October 2024, the national DOD rate had decreased to 24.36 per 100 000 population. The Midwest and Northeast DOD rates receded to prepandemic levels at 21.58 per 100 000 population (lowest rate since 2016) and 21.02 100 000 population (lowest rate since 2015), respectively. Even though the 2024 data are only current through October 2024, subanalyses revealed that DOD rates in 3 states in the West—Nevada, Utah, and Alaska—continued to accelerate.^17^

Table 1 presents DOD rates, and Table 2 shows whether rates were accelerating or decelerating to assess progress even if DOD rates remained positive.^3^ Each cell in Table 1 shows the calendar year DOD rate, and in Table 2, the annual change from the previous year is shown. For example, in 2023, the overall national DOD rate was 31.35 per 100 000 population, a change of −1.04 per 100 000 population from 2022. National trends revealed a plateau in 2018, an acceleration in 2019, and gradual attenuation by 2023. Despite this overall decline, some populations had year-over-year increases in 2023, including adults aged 55 years or older and American Indian or Alaska Native, Black or African American, Hispanic or Latino, Native Hawaiian or Other Pacific Islander, and multiracial individuals. By late 2023, death rates continued to accelerate among adults aged 55 years or older (0.07 per 100 000 population) and American Indian or Alaska Native (0.02 per 100 000 population), Black or African American (1.70 per 100 000 population), Hispanic or Latino (0.20 per 100 000 population), and multiracial (0.28 per 100 000 population) populations, though the pace of increase was slowing, suggesting a potential inflection point.

**Table 1.** DOD Rates by Decedent Demographics

Characteristic	DODs, per 100 000 population^a^								
	2015	2016	2017	2018	2019	2020	2021	2022	2023
Overall	16.34	19.70	21.61	20.62	21.52	27.69	32.13	32.39	31.35
Age, y									
15-24	9.68	12.37	12.65	10.81	11.19	16.49	17.22	15.40	13.50
25-34	26.98	34.52	38.44	35.52	35.64	47.69	52.88	50.58	45.61
35-44	28.45	35.06	39.13	38.32	40.47	52.93	61.97	62.86	60.83
45-54 y	30.16	34.59	37.88	35.33	36.90	46.04	53.76	55.15	53.32
≥55	13.19	15.13	16.49	16.83	17.99	20.54	26.45	27.92	27.99
Race									
American Indian or Alaska Native	NA	NA	NA	16.33	19.15	25.69	34.34	38.43	38.45
Asian	NA	NA	NA	3.37	3.65	4.89	5.13	5.87	5.56
Black or African American	NA	NA	NA	21.19	24.64	34.88	43.73	47.18	48.88
Native Hawaiian or Other Pacific Islander	NA	NA	NA	10.71	10.41	12.09	18.16	17.46	23.23
White	NA	NA	NA	22.38	22.89	28.87	32.91	32.50	30.85
Multiracial	NA	NA	NA	8.33	9.12	13.41	15.56	16.63	16.91
Sex									
Female	11.95	13.47	14.36	13.53	13.66	16.78	19.33	19.09	18.22
Male	20.88	26.13	29.09	27.93	29.62	38.81	45.19	45.96	44.76
Ethnicity									
Hispanic or Latino	7.32	9.10	10.22	10.62	12.34	17.04	20.61	22.06	22.26
Not Hispanic or Latino	18.11	21.83	23.94	22.70	23.43	29.96	34.59	34.52	33.18

Abbreviations: DOD, drug overdose death; NA, not applicable.

^a^ Based on Centers for Disease Control and Prevention WONDER and US Census Bureau intercensal population estimates.

**Table 2.** Annual Change in DOD Rates by Decedent Demographics

Characteristic	Annual change in DOD rate^a^								
	2015	2016	2017	2018	2019	2020	2021	2022	2023
Overall	1.56	3.36	1.91	−0.99	0.90	6.17	34.44	0.26	−1.04
Age, y									
15-24	1.03	2.69	0.28	−1.84	0.38	5.30	0.73	−1.82	−1.90
25-34	3.84	7.54	3.92	−2.92	0.12	12.05	5.19	−2.30	−4.97
35-44	3.37	6.61	4.07	−0.81	2.15	12.46	9.04	0.89	−2.03
45-54 y	1.86	4.43	3.29	−2.55	1.57	9.14	7.72	1.39	−1.83
≥55	0.78	1.94	1.36	0.34	1.16	2.55	5.91	1.47	0.07
Race									
American Indian or Alaska Native	NA	NA	NA	NA	2.82	6.54	8.65	4.09	0.02
Asian	NA	NA	NA	NA	0.28	1.24	0.24	0.74	−0.31
Black or African American	NA	NA	NA	NA	3.45	10.24	8.85	3.45	1.70
Native Hawaiian or Other Pacific Islander	NA	NA	NA	NA	−0.30	1.68	6.07	−0.70	5.77
White	NA	NA	NA	NA	0.51	5.98	4.04	−0.41	−1.65
Multiracial	NA	NA	NA	NA	0.79	4.29	2.15	1.07	0.28
Sex									
Female	0.66	1.52	0.89	−0.83	0.13	3.12	2.55	−0.24	−0.87
Male	2.49	5.25	2.96	−1.16	1.69	9.19	6.38	0.77	−1.20
Ethnicity									
Hispanic or Latino	0.96	1.78	1.12	0.40	1.72	4.70	3.57	1.45	0.20
Not Hispanic or Latino	1.67	3.72	2.11	−1.24	0.73	6.53	4.63	−0.07	−1.34

Abbreviations: DOD, drug overdose death; NA, not applicable.

^a^ Based on Centers for Disease Control and Prevention WONDER and US Census Bureau intercensal population estimates.

DOD rates by age group varied substantially. Rates in all age cohorts increased from 2019 to 2021. Adults aged 35 to 44 years had the highest DOD rate in 2022 (62.86 per 100 000 population), but rates decreased in 2023 to 60.83 per 100 000 population (difference, −2.03 per 100 000 population). By 2023, individuals aged 25 to 34 years showed the steepest year-over-year decline (−4.97 per 100 000 population), while adults aged 55 years or older experienced a ninth consecutive year of increase. Still, the year-over-year increase in this age group slowed from 2021 (difference vs 2020, 5.91 per 100 000 population) to 2023 (difference vs 2022, 0.07 per 100 000 population), suggesting an approaching inflection point.

American Indian or Alaska Native individuals had the highest DOD rate in 2023 (38.45 per 100 000 population), a small increase of 0.02 per 100 000 population from 2022. However, acceleration slowed sharply after peaking at an increase of 8.65 per 100 000 population in 2021 compared with 2020, suggesting a nearing inflection point. Similarly, in 2023, Black or African American individuals had the highest DOD rate of any racial group since 1999^1^ at 48.88 per 100 000 population but the smallest annual increase since 2018, indicating a potential transition. In contrast, Native Hawaiian or Other Pacific Islander individuals showed the largest acceleration in 2023: 23.23 per 100 000 population vs 17.46 per 100 000 population in 2022, with a year-over-year increase of 5.77 per 100 000 population. The DOD rate among multiracial individuals peaked at 16.91 per 100 000 population in 2023, but the annual increase in this group slowed to 0.28 per 100 000 population vs 2022, again signaling a pending reversal. The Hispanic or Latino population showed continuous increases after 2015, but the year-over-year rate of change slowed to 0.20 per 100 000 population in 2023, also suggesting a turning point.

## Discussion

This study found a national deceleration in DOD rates from 2015 to 2024. Although monthly data were noisy, our approach of calculating DOD rates using 12-month moving sums and mid-year intercensal population estimates aimed to reduce volatility while preserving meaningful trends and capturing emerging patterns. Joinpoint analysis identified August 2023 as a statistically significant inflection point when national DOD rates began to decline. While absolute overdose deaths or rates did not peak at this time point, it marked a turning point. Joinpoint analysis distinguished sustained trends (eg, inclines or declines) from short-term fluctuations (eg, spikes or plateaus).

However, this national decline was not evenly distributed. Adults aged 55 years or older and American Indian or Alaska Native, Black or African American, Hispanic or Latino, Native Hawaiian or Other Pacific Islander, and multiracial individuals continued to experience rising DOD rates by the end of 2023. Compared with the national DOD rate peak in October 2023, the peak DOD rate was reached earlier in some regions (ie, Northeast, Midwest, and South), which experienced the shift in opioid supplies to fentanyl-adulterated heroin in 2014.^23^ The West census region, which had low rates in 2015 (likely due to fentanyl’s later arrival^24^), had a later peak. By 2023, the acceleration pattern previously observed in other regions had emerged in the West.^1,17^ Similar delays in drug exposure and treatment may explain racial and ethnic disparities. DOD rates in Black and Latino communities increased more rapidly in the surge between 2019 and 2022, concurrent with lower rates of initiated medication-assisted treatment,^25^ suggesting that the evolving drug supply disproportionately harms racial and ethnic minority populations. Structural inequities and disparities in treatment access likely contribute to this pattern.^13,14^

One plausible explanation for the national DOD rate decline is a cohort effect: individuals most vulnerable to overdose may have already died, currently shrinking the high-risk population. This cohort death effect aligns with this study’s observed surges followed by plateaus or declines. Understanding such mechanisms is critical to sustaining progress and extending DOD rate deceleration to populations still experiencing increases.

While the DOD rate accelerated and decelerated during the onset and waning of the COVID-19 pandemic,^16^ it is important to note that acceleration was already under way by 2019 and early 2020, prior to the pandemic’s emergence.^3^ This early shift is often obscured when using annual data rather than monthly or quarterly trends. Although a recent study highlighted the pandemic’s role in shaping overdose trajectories,^10^ in the current study, the Northeast census region achieved DOD rates lower than those observed in 2017 and the Midwest achieved rates below those from October 2018. This pattern mirrors the temporal progression of the overdose crisis, with the Northeast first experiencing elevated rates in October 2015 and in 2024 reporting the lowest DOD rate among all US regions. In the present analysis, which included 4 additional months of data beyond what was reported by Kiang and Humphreys,^16^ national trends and rates in the South and West remained consistent with the pandemic-related effects described by Kiang and Humphreys, while the Northeast and Midwest appeared to have distinct, region-specific trajectories.

Harm reduction efforts may also play a role. Expanded naloxone access,^26^ shifts in drug use (eg, from injecting to smoking fentanyl),^27^ and changes in drug composition, such as the introduction of adulterants like xylazine,^28,29,30^ may be mitigating overdose risk. These factors warrant further investigation.

Given the association between opioid supply and DOD rates over the past 2.5 decades,^11,12^ a reduction in opioid availability and toxicity may have contributed to the recent decline. A negative supply shock of fewer or less-potent opioids may have reduced deaths. Supporting this theory, opioids in this study accounted for a smaller share of overdose deaths, and opioid-involved DOD rates decelerated more rapidly than stimulant-involved DOD rates, suggesting different causal pathways and intervention opportunities by substance type.

Our study provides evidence of the timing and magnitude of the national DOD rate decline and identifies varying patterns among demographic and geographic subpopulations. These findings enhance the ability to link policy and environmental shifts to overdose trends and may help target interventions to regions and populations still in crisis. However, continued gaps in timely, reliable drug supply data limit our understanding of supply-driven fluctuations in DOD rates. Improving these data systems is essential for more effective prevention and targeted interventions.

In addition, we caution against complacency. While our findings are encouraging, current DOD rates remain at crisis levels above any reasonable threshold for a disease outbreak. Sustained, evidence-based public health responses remain essential to preventing future overdose deaths.

### Limitations

This study has limitations. It relied on provisional data from the NVSS and CDC WONDER, both maintained by the National Center for Health Statistics. NVSS data have an approximate 5-month reporting lag, while WONDER data lag by about 6 months. Both sources may have incomplete case ascertainment. Additionally, underlying cause-of-death data are susceptible to misclassification, or “garbage coding.” For example, a coroner may list respiratory arrest as the cause of death when the actual cause was fentanyl overdose, with respiratory arrest as a symptom. Despite these limitations, provisional surveillance data provide a reasonable proxy for tracking the expansion and contraction of the drug overdose crisis. While monthly DOD rates can be estimated nationally and regionally through October 2024, the absence of monthly intercensal demographic estimates restricts more granular demographic analyses to calendar year data.

## Conclusions

This cross-sectional study found that the US experienced a significant deceleration in DODs from 2015 through 2024. However, this trend was not uniform across geographic regions, demographic subpopulations, or drug types. Further research is essential to sustain progress in DOD reduction and ensure all populations benefit.

## Notes

Supplement.
Data Sharing Statement
