# Supplementary material for: Decline in US Drug Overdose Deaths by Region, Substance, and Demographics
Source: JAMA Netw Open. 2025 Jun 12;8(6):e2514997. doi: 10.1001/jamanetworkopen.2025.14997 (PMC12163676; doi:10.1001/jamanetworkopen.2025.14997)
Supplement: Supplement. — Data Sharing Statement [file jamanetwopen-e2514997-s001.pdf]

Data Sharing Statement

Post. Decline in US Drug Overdose Deaths by Region, Substance, and Demographics. *JAMA Netw Open*. Published June 12, 2025. doi:10.1001/jamanetworkopen.2025.14997

Data
Data available: Yes
Data types: Other (please specify)
Additional Information: All data used in this analysis is publicly avaiable.
How to access data: Data are compiled from cdc.gov and census.gov data. We will also provide our data file upon request.
When available: With publication

Supporting Documents
Document types: None

Additional Information
Who can access the data: The data is publicly available.
Types of analyses: The data is publicly available.
Mechanisms of data availability: The data is publicly available no support necessary.
Any additional restrictions: No restrictions
